# Supplementary material for: Enhancing the accuracies by performing pooling decisions adjacent to the output layer
Source: Sci Rep. 2023 Aug 31;13:13385. doi: 10.1038/s41598-023-40566-y (PMC10471572; doi:10.1038/s41598-023-40566-y)
Supplement: Supplementary file 1 — Supplementary Information. [file 41598_2023_40566_MOESM1_ESM.pdf]

Supplementary Information

Enhancing the accuracies by performing pooling decisions adjacent to the output layer

Yuval Meir[1], Yarden Tzach[1], Ronit D. Gross[1], Ofek Tevet[1], Roni Vardi[2] and Ido Kanter[1,2,*]

[1]Department of Physics, Bar-Ilan University, Ramat-Gan, 52900, Israel.

[2]Gonda Interdisciplinary Brain Research Center, Bar-Ilan University, Ramat-Gan, 52900, Israel.

[*]Corresponding author email: ido.kanter@biu.ac.il

Advanced VGGm architectures. The examined advanced VGGm (A-VGGm) architectures consist of m layers, $6 \leq m \leq 16$ (Fig. 1A-B in the main text, exemplifies $m = 16$ for VGG16[1] and A-VGG16.

For $m = 6$ and 8, the architecture is similar to the VGG8[1], with initial depth of 64 for the first CL and doubling depth for the next three CLs, and with a single zero-padding around the input of each CL. For $m = 6$, a (2×2) average pooling (AP) is applied after the third CL and an (8×8) max-pooling (MP) after the fifth CL. For $m = 8$, a (2×2) AP is applied after the third CL and a (4×4) MP after the fifth CL. $m = 6$ terminates with one FC layer consisting of 2048 hidden units and $m = 8$ with three FC layers with 8192 hidden units each.

For $m = 14$ and 16,there are 13 CLs with doubling depth (except for the last 3 CLs) and with a single zero-padding around the input of each CL, followed by one FC layer with 8192 hidden units for $m = 14$ and three FC layers with 4096 hidden units for $m = 16$. For both $m = 14$ and 16, a (4×4) AP is applied after the 7th CL and a (2×2) MP is applied after the 13th CL.

For $m = 13$ the last three CLs are withdrawn, resulting in ten CLs, where a (4×4) AP is also applied after the 7th CL and a (4×4) MP is applied after the 10th CL terminating with three FC layers consisting of 2048 hidden units each.

After each CL, a batch normalization layer was applied. The softmax function was applied to the ten outputs. The ReLU activation function was assigned to each hidden unit (not including the ten output units and pooling operators), and all weights were initialized using a uniform distribution with a zero mean and unity standard deviation (Std) according to the He normal initialization[3].

For A-VGG13 and A-VGG16 with linear activation functions for the FC layer the architectures remain the same.

Advanced LeNet5 architectures. The advanced LeNet5 (A-LeNet5) architectures consist of two consecutive CLs of size (5×5) with depths $\mathrm{d}_1 = 6$ and $\mathrm{d}_2 = 16$ and three FC layers (Fig. 3A in the main text). These architectures are similar to the LeNet5[2], however, the pooling operators are applied only after the second CL (Fig. 3A in the main text). The ReLU activation function was assigned to each hidden unit where the softmax function was applied to the ten output units. All weights were initialized

using a uniform distribution with a zero mean and unity Std according to the He normal initialization[3].

Data preprocessing. Each input pixel of an image (32×32) from the CIFAR-10 database was divided by the maximal pixel value, 255, multiplied by 2, and subtracted by 1, such that its range was $[-1, 1]$. In all simulations, data augmentation was used, derived from the original images, by random horizontally flipping and translating up to four pixels in each direction.

Optimization. The cross-entropy cost function was selected for the classification task and was minimized using the stochastic gradient descent algorithm[4,5]. The maximal accuracy was determined by searching through the hyper-parameters (see below). Cross-validation was confirmed using several validation databases, each consisting of $10{,}000$ random examples from the training set, as in the test set. The averaged results were in the same Std as the reported average success rates. The Nesterov momentum[3] and L2 regularization method[4] were applied.

Hyper-parameters. The hyper-parameters η (learning rate), μ (momentum constant[3]), and α (regularization L2[4]) were optimized for offline learning, using a mini-batch size of 100 inputs. The learning rate decay schedule[5,6] was also optimized such that it was multiplied by the decay factor, q, every $\Delta\mathrm{t}$ epochs, and is denoted below as $(\mathrm{q}, \Delta\mathrm{t})$.

Out of phase scheduling. For A-VGG16 and A-VGG8 the decay schedules[5,6] of the FC layers and the CLs learning rates had a phase of 10 epochs in between. The decay scheduling for the FC layers starts at $epoch = 10$, while for the CLs at $epoch = 20$. Specifically, decay learning rate of the FC layers occurs at $epochs = [10, 30, 50\ \ldots]$, while for the CLs at $epochs = [20, 40, 60 \ldots]$.

Detailed A-VGGm Hyper-parameters for Fig. 1 in the main text.

**A-VGG16**				
Layer	η	μ	α	epochs
CLs	0.00721	0.98	1.15e-3	280
FC layers	0.0045	0.982	1.35e-3	280

The decay schedule for the learning rate is defined as follows:

For CLs:

$$(q, \Delta t) = \begin{cases} (0.65,20) & \text{epoch} \leq 140 \\ (0.55,20) & \text{epoch} > 140 \end{cases}$$

For FC layers, with 10 epochs out of phase:

$$(q, \Delta t) = \begin{cases} (0.65,20) & \text{epoch} < 150 \\ (0.5,20) & \text{epoch} \geq 150 \end{cases}$$

The accuracies' Std is 0.0015.

**A-VGG14**				
Layers	η	μ	α	epochs
CLs	0.0078	0.985	1.15e-3	200
FC layers	6.05e-4	0.98	1.15e-3	200

The decay schedule for the learning rate is defined as follows:

For CLs:

$$(q, \Delta t) = (0.65,20)$$

For FC layers:

$$(q, \Delta t) = \begin{cases} (0.55,10) & \text{epoch} < 120 \\ (0.5,10) & \text{epoch} \geq 120 \end{cases}$$

The accuracies' Std is 0.00092.

**A-VGG13**				
Layers	η	μ	α	epochs
CLs	0.0078	0.98	1.15e-3	200
FC layers	0.00297	0.985	1.15e-3	200

The decay schedule for the learning rate is defined as follows:

For CLs:

$$(q, \Delta t) = (0.65, 20)$$

For FC layers:

$$(q, \Delta t) = \begin{cases} (0.55,20) & \text{epoch} < 120 \\ (0.5,20) & \text{epoch} \geq 120 \end{cases}$$

The accuracies' Std is 0.0012.

A-VGG8				
Layers	η	μ	α	epochs
CLs	0.0145	0.97	1e-3	200
FC layers	0.002	0.975	1.2e-3	200

The decay schedule for the learning rate is defined as follows:

For CLs:

$$(q, \Delta t) = \begin{cases} (0.66,20) & \text{epoch} \leq 140 \\ (0.55,20) & \text{epoch} > 140 \end{cases}$$

For FC layers with 10 epochs out of phase:

$$(q, \Delta t) = \begin{cases} (0.66,20) & \text{epoch} < 150 \\ (0.5,20) & \text{epoch} \geq 150 \end{cases}$$

The accuracies' Std is 0.0009.

A-VGG6				
Layers	η	μ	α	epochs
CLs	9.75e-3	0.972	1.1e-3	200
FC layers	1.95e-3	0.98	1.1e-3	200

The decay schedule for the learning rate is defined as follows:

For CLs:

$$(q, \Delta t) = \begin{cases} (0.65,20) & \text{epoch} < 120 \\ (0.55,20) & \text{epoch} \geq 120 \end{cases}$$

For FC layers:

$$(q, \Delta t) = \begin{cases} (0.65, 20) & \text{epoch} < 120 \\ (0.5, 20) & \text{epoch} \geq 120 \end{cases}$$

The accuracies' Std is 0.00224.

A-VGG13 and A-VGG16 with linear activation functions for the FC layers.

A-VGG16 with linear activation				
Layer	η	μ	α	epochs
CLs	0.0078	0.98	1.15e-3	200
FC layers	0.00297	0.985	1.15e-3	200

The decay schedule for the learning rate is defined as follows:

For CLs:

$$(q, \Delta t) = (0.65, 20)$$

For FC layers:

$$(q, \Delta t) = \begin{cases} (0.55, 20) & \text{epoch} < 120 \\ (0.5, 20) & \text{epoch} \geq 120 \end{cases}$$

The accuracies' Std is 0.0014.

A-VGG13 with linear activation				
Layer	η	μ	α	epochs
CLs	0.0078	0.98	1.15e-3	200
FC layers	0.00297	0.985	1.15e-3	200

The decay schedule for the learning rate is defined as follows:

For CLs:

$$(q, \Delta t) = (0.65, 20)$$

For FC layers:

$$(q, \Delta t) = \begin{cases} (0.55, 20) & \text{epoch} < 120 \\ (0.5, 20) & \text{epoch} \geq 120 \end{cases}$$

The accuracies' Std is 0.001.

Detailed explanations for the main text Fig. 2. In Fig. 2 in the main text, two architectures were compared, each consists of ten CLs with unity depth and the same ten (3×3) filters. Random inputs of size (1024×1024) with values taken from a Gaussian distribution with zero mean and unity Std were tested. The ReLU activation function was assigned to all the hidden and output units.

Two architectures were compared, sequence pooling (SP) and top pooling (TP). The SP architecture consists of (2×2) MP after each one of the first n CLs, whereas for the TP architecture $(2^n\times 2^n)$ MP is applied after the 10th CL (Fig. 2B top in the main text, exemplifies $n=4$). For a given $n=[2,4,6,8,10]$, the $(2^{10-n}\times\ 2^{10-n})$ SP and TP output ratios, $\frac{O_{SP}}{O_{TP}}$, were calculated and the probability $P\left(\frac{O_{SP}}{O_{TP}}>1\right)$ was estimated using 20,000 - 100,000 different random inputs (depending on n), and filters which were randomly initialized for the CLs on each sample.

The increase in CLs depth beyond unity does not qualitatively affect $P\left(\frac{O_{SP}}{O_{TP}}>1\right)$, as indicated by simulations of VGG8 with five consecutive (2×2) MP operators after each CL and a single (32×32) MP after five CLs. The same five random (3×3) convolutions were used for both architectures, and the 512 ratios, $\frac{O_{SP}}{O_{TP}}$, for the single output of each filter, were calculated. The probability $P\left(\frac{O_{SP}}{O_{TP}}>1\right)$ was calculated by averaging over randomly selected batches of CIFAR10 training inputs and several selected sets of fixed random convolutions.

Detailed A-LeNet5 Hyper-parameters for Fig. 3 in the main text.

**A-LeNet5 - a**			
η	μ	α	epochs
0.032	0.92	5e-4	240

The decay schedule for the learning rate is defined as follows:

$$(\mathrm{q},\Delta\mathrm{t})=\begin{cases}(0.8,10) & \text{epoch}<120\\ (0.7,10) & \text{epoch}\geq 120\end{cases}$$

The accuracies' Std is 0.0028.

A-LeNet5 - b			
η	μ	α	epochs
0.03	0.93	4e-4	280

The decay schedule for the learning rate is defined as follows:

$$(q, \Delta t) = \begin{cases} (0.8,10) & \text{epoch} < 120 \\ (0.7,10) & \text{epoch} \geq 120 \end{cases}$$

The accuracies' Std is 0.003.

A-LeNet5 - c			
η	μ	α	epochs
0.028	0.925	5e-4	280

The decay schedule for the learning rate is defined as follows:

$$(q, \Delta t) = \begin{cases} (0.8,10) & \text{epoch} < 120 \\ (0.7,10) & \text{epoch} \geq 120 \end{cases}$$

The accuracies' Std is 0.0025.

A-LeNet5 - d			
η	μ	α	epochs
0.032	0.92	5e-4	240

The decay schedule for the learning rate is defined as follows:

$$(q, \Delta t) = \begin{cases} (0.8,10) & \text{epoch} < 120 \\ (0.7,10) & \text{epoch} \geq 120 \end{cases}$$

The accuracies' Std is 0.0035.

A-LeNet5 - e			
η	μ	α	epochs
0.02	0.922	1.2e-3	240

The decay schedule for the learning rate is defined as follows:

$$(\mathrm{q}, \Delta \mathrm{t}) = \begin{cases} (0.8,10) & \text{epoch} < 120 \\ (0.7,10) & \text{epoch} \geq 120 \end{cases}$$

The accuracies' Std is 0.0039.

Statistics. Statistics for each architecture were obtained using 10 samples.

Hardware and software. We used Google Colab Pro and its available GPUs. We used Pytorch for all the programming processes.

1. Simonyan, K. & Zisserman, A. Very Deep Convolutional Networks for Large-Scale Image Recognition. (2014).

2. LeCun, Y. *et al.* Backpropagation Applied to Handwritten Zip Code Recognition. *Neural Comput* **1**, 541–551 (1989).

3. Botev, A., Lever, G. & Barber, D. Nesterov's accelerated gradient and momentum as approximations to regularised update descent. in *2017 International Joint Conference on Neural Networks (IJCNN)* 1899–1903 (IEEE, 2017). doi:10.1109/IJCNN.2017.7966082.

4. Schmidhuber, J. Deep learning in neural networks: An overview. *Neural Networks* **61**, 85–117 (2015).

5. He, K., Zhang, X., Ren, S. & Sun, J. Deep Residual Learning for Image Recognition. (2015).

6. You, K., Long, M., Wang, J. & Jordan, M. I. How Does Learning Rate Decay Help Modern Neural Networks? (2019).
